# Hahn-PCNN-CNN: an end-to-end multi-modal brain medical image fusion framework useful for clinical diagnosis

**DOI:** 10.1186/s12880-021-00642-z

**Published:** 2021-07-14

**Authors:** Kai Guo, Xiongfei Li, Xiaohan Hu, Jichen Liu, Tiehu Fan

**Affiliations:** 1 Key Laboratory of Symbolic Computation and Knowledge Engineering of Ministry of Education, Jilin University, Changchun, China; 2 College of Computer Science and Technology, Jilin University, Changchun, China; 3 Department of Radiology, The First Hospital of Jilin University, Changchun, China; 4 College of Software, Jilin University, Changchun, China; 5 College of Instrumentation and Electrical Engineering, Jilin University, Changchun, China

**Keywords:** Multi-modal brain medical image, Brain medical image fusion, Clinical diagnosis, Deep learning, Hahn moment

## Abstract

**Background:**

In medical diagnosis of brain, the role of multi-modal medical image fusion is becoming more prominent. Among them, there is no lack of filtering layered fusion and newly emerging deep learning algorithms. The former has a fast fusion speed but the fusion image texture is blurred; the latter has a better fusion effect but requires higher machine computing capabilities. Therefore, how to find a balanced algorithm in terms of image quality, speed and computing power is still the focus of all scholars.

**Methods:**

We built an end-to-end Hahn-PCNN-CNN. The network is composed of feature extraction module, feature fusion module and image reconstruction module. We selected 8000 multi-modal brain medical images downloaded from the Harvard Medical School website to train the feature extraction layer and image reconstruction layer to enhance the network’s ability to reconstruct brain medical images. In the feature fusion module, we use the moments of the feature map combined with the pulse-coupled neural network to reduce the information loss caused by convolution in the previous fusion module and save time.

**Results:**

We choose eight sets of registered multi-modal brain medical images in four diease to verify our model. The anatomical structure images are from MRI and the functional metabolism images are SPECT and 18F-FDG. At the same time, we also selected eight representative fusion models as comparative experiments. In terms of objective quality evaluation, we select six evaluation metrics in five categories to evaluate our model.

**Conclusions:**

The fusion image obtained by our model can retain the effective information in source images to the greatest extent. In terms of image fusion evaluation metrics, our model is superior to other comparison algorithms. In terms of time computational efficiency, our model also performs well. In terms of robustness, our model is very stable and can be generalized to multi-modal image fusion of other organs.

## Background

Deep learning technology is currently revolutionizing medical diagnostic services. Convolutional networks are fusing or surpassing human operators in multi-modal brain medical image fusion and are increasingly proposed as an aid to human medical decision-making. Multi-modal brain medical images use different sensors to image the head to show the anatomy and metabolism of the head [1–3]. Among them, Computed Tomography (CT) and Magnetic Resonance Imaging (MRI) display the structural information of organs with high spatial resolution. They are called structural images. Positron Emission Tomography (PET) and Functional Magnetic Resonance Imaging (fMRI) images provide information about the function of organs. They are called functional images. Each unimodal image has its own characteristics. CT images are used to clearly show bone structure information, while MRI images are good at showing the physiological details of soft tissues. PET images can be used to quantitatively and dynamically detect human metabolites or drugs, while fMRI images are good at detecting changes in blood flow in the magnetic field of brain cells to help confirm the diagnosis. For example, glioma is the most common brain tumor [4–6], accounting for 80% of all malignant brain tumors [7]. The symptoms are not only related to their metabolism in the functional image, but also have an important relationship with their position in the brain. Therefore, it is necessary to perform multi-mode medical image fusion to gather all the features of multi-source images into an image with high contrast and resolution [8–12]. The obtained fusion image not only helps doctors make a more favorable diagnosis of patients, but also reduces the uncertainty of medical images generated by multiple sensors [13–15].

We will explain with a detailed example below.

**Fig. 1:**
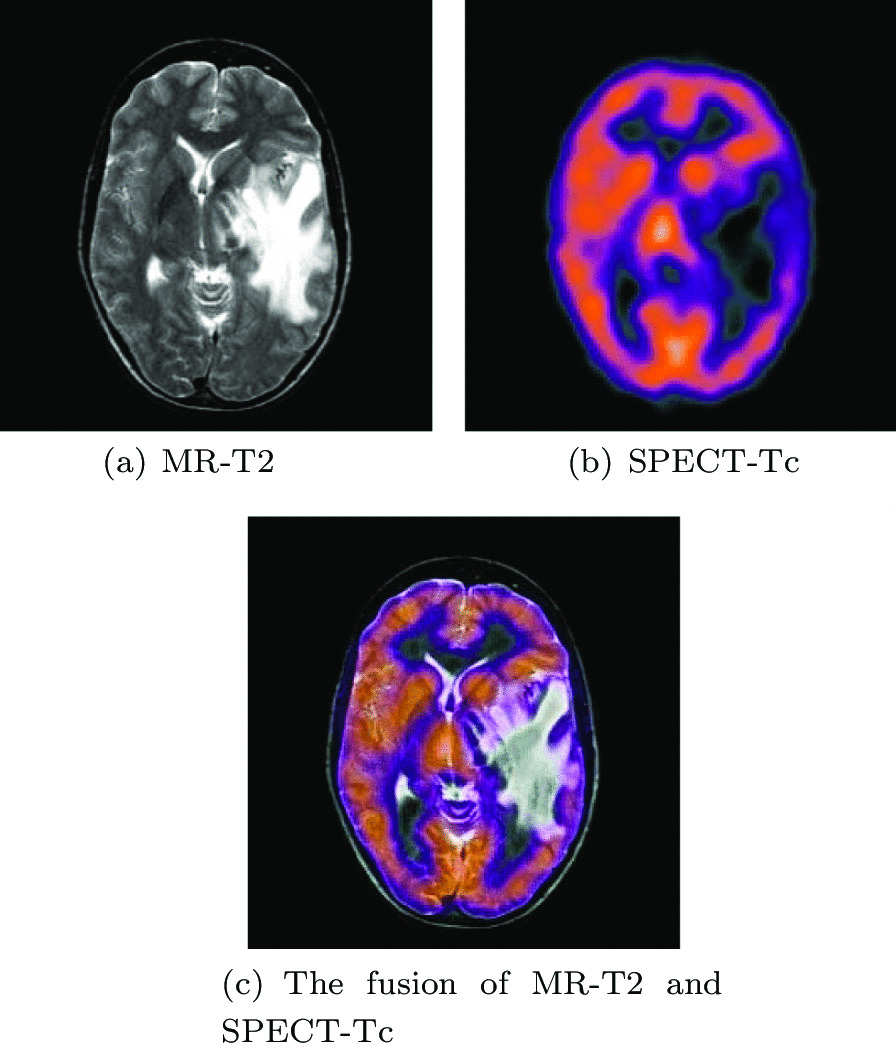
Multi-modal image of a brain metastasis of a bronchial cancer

As shown in Fig. 1, brain images showed a large mass with edema around it. The mass and edema pressure forced the midline to shift and adjacent midbrain structures were compressed. On the Proton Density (PD) and T2-weighted (T2) images of Magnetic Resonance (MR), the large area of the left temporal area showed high signal intensity. On the contrast-enhanced image, the lesion contains cystic components. A narrower sulcus in the left cerebral hemisphere indicates severe swelling of the left cerebral hemisphere. In addition, according to perfusion Single-Photon Emission Computed Tomography (SPECT) imaging, the blood flow in the lesion area is very low. It can be seen that the fused image can reduce the diagnosis scope and eliminate interference information better than the unfused source image.

At this stage, the mainstream algorithms that prevail in multi-modal brain medical images are generally divided into two categories, namely filtering hierarchical fusion algorithms and deep learning algorithms. Among them, Dual-Tree Complex Wavelet Transform (DTCWT) [16] algorithm and Nonsubsampled Contourlet (NSCT) algorithm [17] are representative filtering hierarchical fusion algorithms. In deep learning, Coupled Neural Network (CNN) [18] algorithm and Laplacian Pyramid Sparse Representation (LPSR) algorithm [19] are more typical. Of course, there are Guided Filtering Fusion (GFF) algorithm [20], Internal Generative Mechanism (IGM) algorithm [21], Visual Saliency Map and Weighted Least Square (VSMWLS) algorithm [22]and Laplacian Re-Decomposition (LRD) algorithm [23] in between are also algorithms with good performance.

Although the above eight algorithms have achieved good fusion results, they also have their own shortcomings. The traditional algorithm pyramid decomposition wavelet transform fusion algorithm has low time complexity, however, the overall brightness of the fusion image is dark and some areas have inexplicable shadows, which shows that their details are mishandled; the image fidelity and color saturation of the convolutional neural network fusion are very good, which shows its ability to extract features. Of course, the finer the feature extraction, the higher the time complexity. At the same time, the more feature extraction, the easier it is to cause artifacts in the fusion image. In order to better express the tissue metabolism, the remaining algorithm enhances the brightness of the fusion image without changing the color information, resulting in low image contrast and affecting the presentation of the image structure. Based on the problems of the above algorithms, we propose a fusion model. The model selects the convolutional neural network to construct the feature extraction module and the image reconstruction module. In the feature fusion module, Hahn moments are used to guide the potential block to activate Pulse-Coupled Neural Network (PCNN) to realize feature map fusion. Convolutional neural networks are currently the best technology in the field of feature extraction and image reconstruction. PCNN is a global fusion algorithm that can retain more detailed information, and its signal form and processing principles are more in line with the physiological basis of the human visual nervous system . In solving the blurring of image structure, Hahn moments can represent the shape of the image well. As for how to avoid fusion artifacts, we introduce the Hahn moment energy of the block.

## Methods

### Related work

#### Hahn moment

The moment invariants of the image are good at describing the characteristics of the image. It is not only simple, but also immune to interference from light, noise and geometric distortion. Among these moments, the Hahn moment is widely used because it has a more general meaning. The Hahn moment is a kernel function of the orthogonal polynomial of Hahn. The Hahn polynomial of order N can be expressed as

1 $$\begin{aligned} h_n^{(\alpha ,\beta )}\left( x \right)= & {} \frac{{{B_n}}}{{\rho \left( x \right) }}{\nabla ^n}\left[ {{\rho _n}\left( x \right) } \right] \end{aligned}$$

2 $$\begin{aligned} {\nabla ^n}f\left( x \right)= & {} \sum \limits _{k = 0}^n {{{( - 1)}^k}\frac{{n!}}{{k!(n - k)!}}f(x - k)} \end{aligned}$$

where $x,n = 0,1,2, \ldots N - 1$, *N* is a given positive integer. $\alpha > - 1$, $\beta > - 1$, ${B_n}(x) = \frac{{{{( - 1)}^n}}}{{n!}}$, $\rho (x) = \frac{{\Gamma (N + \alpha - x)\Gamma (\beta + 1 + x)}}{{\Gamma (x + 1)\Gamma (N - x)}}$ and ${\rho _n}(x) = \frac{{\Gamma (N + \alpha - x)\Gamma (n + \beta + 1 + x)}}{{\Gamma (x + 1)\Gamma (N - n - x)}}$.

In order to reduce the oscillation in the calculation of higher moments and avoid the divergence of polynomial values, the polynomial is regularized. The regularized Hahn polynomial is defined as

3 $$\begin{aligned} {\overline{h}} _n^{(\alpha ,\beta )}(x) = h_n^{(\alpha ,\beta )}(x)\sqrt{\frac{{\rho (x)}}{{d_n^2}}} \end{aligned}$$

where $x,n = 0,1,2, \ldots N - 1$ and $d_n^2 = \frac{{\Gamma (\alpha + n + 1)\Gamma (\beta + n + 1)\Gamma (\alpha + \beta + n + 1 + N)}}{{(\alpha + \beta + 2n + 1)n!(N - n - 1)!\Gamma (\alpha + \beta + n + 1)}}$. Hahn moments of order $(n + m)$ in terms of weighted Hahn polynomials, for an image $(M \times N)$ with intensity function *f*(*x*, *y*) is defined as

4 $$\begin{aligned} {H_{mn}} = \sum \limits _{x = 0}^{N - 1} {\sum \limits _{y = 0}^{M - 1} {{\overline{h}} _m^{(\alpha ,\beta )}(x)} } {\overline{h}} _n^{(\alpha ,\beta )}(y)f(x,y) \end{aligned}$$

where $x,y \in [0,N - 1]$. The parameters *N* and *M* are substituted with *N-1* and *M-1* respectively to match the pixel points of an image.

#### Simplified PCNN

In view of the fact that many parameters of the original PCNN require feedback iteration to adjust, which is both inconvenient and time complexity. In this article, we use a simplified PCNN neuron model [24]. This model is loved by most scholars, so it is widely used. Its mathematical model is described as follows:

5 $$\begin{aligned} {F_{ij}}(n)= & {} {S_{ij}} \end{aligned}$$

6 $$\begin{aligned} {L_{ij}}(n)= & {} \sum {{W_{ijkl}}{Y_{ij}}} \end{aligned}$$

7 $$\begin{aligned} {U_{ij}}(n)= {F_{ij}}(1 + \beta {L_{ij}}(n)) \end{aligned}$$

8 $${T_{ij}}(n)= {e^{ - {\alpha _T}}}{T_{ij}}(n - 1) + {V_T}{Y_{ij}}(n - 1)$$

9 $${Y_{ij}}(n)= \left\{ {\begin{array}{*{20}{c}} {1,{U_{ij}}(n) \ge {\theta _{ij}}(n)}\\ {0,{U_{ij}}(n) < {\theta _{ij}}(n)} \end{array}} \right.$$

In simplified PCNN, the input neuron in the *F* channel is the pixel gray value of the external image, and the input neuron in the *L* channel only considers the output of the internal neuron affected by the eight neighborhoods. This operation greatly reduces the time complexity.

When the $P \times Q$ image is input into PCNN, PCNN becomes a network composed of $P \times Q$ neurons. The gray value of each input pixel is each stimulation signal ${S_{ij}}$. When there are pixels with similar gray values in the neighborhood of the inner matrix W and M, the pulse output generated by stimulating a pixel will cause the stimulation of the corresponding neuron in the neighborhood of the pixel with similar gray value, resulting in an edge including edge, Pulse output sequence of texture and area information. The binary image produced by it is a fusion image, so the parameters of PCNN affect the result of image fusion.

### The framework of our multi-modal brain medical image fusion

**Fig. 2:**
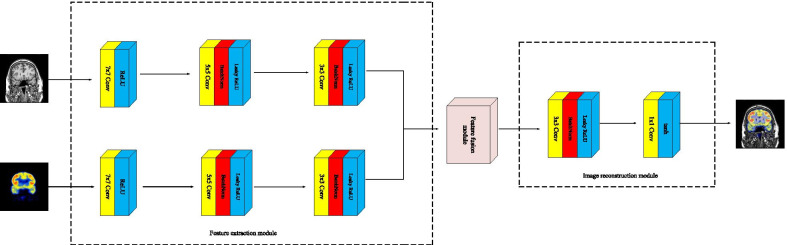
Our multi-modal brain medical image fusion model

In terms of image processing, the convolutional layer can extract features, which are usually more meaningful and valuable than the features obtained by traditional feature extractors. In addition, the convolutional layer also acts as a weighted average to generate output images. We design a multi-modal brain medical image fusion framework based on CNN with Hahn moment, hereinafter referred to as Hahn-PCNN-CNN. Hahn-PCNN-CNN consists of feature extraction module, feature fusion module and image reconstruction module. As shown in Fig. 2, the feature extraction module is employed to extract image information features from MR-T1 and Positron Emission Tomography labeling Glucose with Positron Nuclide 18F (18F-FDG). Secondly, the convolution feature of source images is fused through the feature fusion module. Finally, the image reconstruction module is used to generate the fused image.

### Feature extraction module

In the feature extraction module, we intend to use three convolutional layers to extract image features from source images. Feature extraction is a key step in the transform domain image fusion algorithm. For CNN, the stochastic initialization convolution kernel training regression model has been faced with instability problem and it is difficult to train. The best approach is to take advantage of model migration in migration learning. Therefore, we plan to use the first convolutional layer of GoogLeNet, which is pre-trained on ImageNet, as the First Convolutional Layer (CONV1) of our network , where it contains 64 convolution kernels of $7 \times 7$ size, which can extract many effective features of the image.

Although CONV1 has a wide acceptance range, more graphics functions can be used. Therefore, not all image features must be displayed in the fusion. The key to multi-mode brain medical image fusion is the fusion of effective information, that is, while giving clear edges to metabolic information, as much as possible to ensure that the texture of the structural image is clear, so as to help doctors make a correct diagnosis. Therefore, the Second Convolutional Layer (CONV2) and the Third Convolutional Layer (CONV3) are added to filter the features obtained by CONV1 and finally obtain a feature map that can be fused.

If the image is undersampled, part of the image information will be lost. Therefore, in the feature selection module, we do not want to lose the information of the image. Adjust the value of the corresponding stride and padding for the kernel of each convolution layer. Details of each convolutional layer of feature extraction module are shown in the Table 1.

**Table 1:** Details of each convolutional layer of feature extraction module

Layer	Setting			
	Kernel number	Convolution kernel	Stride	Padding
CONV 1	64	$7 \times 7$	1	3
CONV 2	64	$5 \times 5$	1	2
CONV 3	64	$3 \times 3$	1	1

### Feature fusion module

We input the feature maps that are obtained from CONV3 into the image fusion module. Firstly, divide them into blocks and calculate the Hahn moment of each block. Then, inspired by the expansion coefficient factor, we defined the potential of the image patch. Finally, we use the potential of the image block as the external stimulus of PCNN to achieve the fusion of feature maps.

The size of a brain medical image I(x,y) is $M \times N$. It will be divided into blocks of the same size; the block size is $D \times D$. n order to get a block of integers, we choose to fill with the number 0 so that M and N are equal. After the fusion is completed, the filled elements are removed to obtain the fused image. All the divided blocks will form a set called $\{ C_{ij}^I\}$, $i \in \left\{ {1,2,3, \ldots ,M/D} \right\}$, $j \in \left\{ {1,2,3, \ldots ,N/D} \right\}$. Hahn moments of image blocks of $\{ C_{ij}^I\}$ can be expressed as

10 $$\begin{aligned} {H_{ij}} = \left[ {\begin{array}{*{20}{c}} {{H_{11}}}&{}{{H_{12}}}&{} \cdots &{}{{H_{1n}}}\\ {{H_{21}}}&{}{{H_{22}}}&{} \cdots &{}{{H_{2n}}}\\ \vdots &{} \vdots &{} \ddots &{} \vdots \\ {{H_{m1}}}&{}{{H_{m2}}}&{} \cdots &{}{{H_{mn}}} \end{array}} \right] \end{aligned}$$

where $m,n \in \left\{ {1,2,3, \ldots ,D} \right\}$, The Potential Energy (*PE*) of Hahn moments values is computed by

11 $$\begin{aligned} P{E_{uv}} = \sum \limits _{u = 1}^m {\sum \limits _{v = 1}^n {\frac{{\left| {{H_{uv}} - {H_{11}}} \right| }}{{\sqrt{{{\left( {u - 1} \right) }^2} + {{\left( {v - 1} \right) }^2}} }}} } \end{aligned}$$

where $u \in \left\{ {1,2, \ldots ,m} \right\}$, $v \in \left\{ {1,2, \ldots ,n} \right\}$. Its structure is based on the principle of linear expansion coefficient in physics.

In image fusion, how to make full use of the biological features of PCNN and combine PCNN attributes and image characteristics to determine relevant parameters has always been a research hotspot in PCNN adaptive image fusion. PCNN has a strong adaptive link strength and will not change due to changes in the image. Humans have always liked to judge the clarity of the image based on the visually significant area. Whether it is a functional image or a structural image, they all have a prominent area to express information and an inconspicuous black background. And we tend to pay more attention to the former. After all, the visually significant areas are clear. Visual saliency detection through image complexity function is introduced into multi-modal brain medical image fusion. In view of its good performance, it can detect the saliency of medical images well. In this article, the visually significant areas detected by the Complexity-Weighted Saliency (CWS) detection [25] model are used as the link strength of the corresponding neurons. When the link strength beta and the external stimulus *PE* are determined, they are both input into the pulse coupled neural network. The feature map fusion starts according to the following formula:

12 $${T^A}= PCNN(PE_{uv}^A)$$

13 $${T^B}= PCNN(PE_{uv}^B)$$

14 $$\begin{aligned} F= & {} \left\{ {\begin{array}{*{20}{c}} {\max (A(i,j),B(i,j))\mathrm{{ }}{T^A} = {T^B}}\\ {A(i,j){T^A} > {T^B}}\\ {B(i,j){T^A} < {T^B}} \end{array}} \right. \end{aligned}$$

where *A* represents the structure image and *B* represents the functional image. *F* represents the fused images. $PE_{uv}^A$ and $PE_{uv}^B$ are external stimuli of PCNN. The linking strength ${\beta ^A}$ and ${\beta ^B}$ are calculated by the CWS model. The firing times matrix ${T^A}$ and ${T^B}$ are determined by ${\beta ^A}$ and ${\beta ^B}$, respectively.

### Image reconstruction module

Since our feature extraction module is composed of three convolutional layers, we continue to use the convolutional layer to reconstruct it to get our final fused image after the feature map is fused. In the image reconstruction module, we choose two convolutional layers, namely CONV4 and CONV5. The convolution kernel of CONV4 is 3 × 3, stride and padding are both set to 1. The convolution kernel of CONV5 is 1 × 1, stride and padding are both set to 0. The kernel number of CONV4 is the same as CONV3. Since CONV5 reconstructs feature maps into a three-channel output. Thereby, the kernel number of CONV5 is 3. Details of each convolutional layer of the image reconstruction module are shown in the Table 2.

**Table 2:** Details of each convolutional layer of image reconstruction module

Layer	Setting			
	Kernel number	Convolution kernel	Stride	Padding
CONV 4	64	$3 \times 3$	1	1
CONV 5	3	$1 \times 1$	0	0

### Loss function

All researchers who use neural networks for image fusion will face the problem of information loss. Therefore, how to reduce information loss is particularly important. The loss function that determines the amount of information loss has become the focus of research. In the case of ground-truth image, Mean Square Error (MSE) is generally used as a loss function and the objective function is to reduce the distortion of images. However, multi-modal brain medical image fusion has two characteristics that make it unable to use MSE as a loss function. One is that there is no reference image for brain medical images and the other is that brain medical images are multi-modal. Therefore, the fusion image should retain the texture, edge and metabolic activity information in the source image as much as possible, not simply inherit all the information in the source image.

In the article, we plan to use cross_entropy loss function, Multiscale-Structural Similarity (MS-SSIM) loss function and Total Variation (TV) loss function. Cross_entropy loss function can reduce the loss in the pixel model pixel by pixel; MS-SSIM loss function can reduce the loss of brightness, contrast and structural information in the reconstruction; TV loss function can make our images smoother while eliminating noise in the source image. The total loss function formula is as follows.

15 $$\begin{aligned} {L_{total}} = {L_{cross\_entropy}} + {L_{MS - SSIM}} + {L_{TV}} \end{aligned}$$

Cross_entropy loss function can be described as

16 $$\begin{aligned} {L_{cross\_entropy}} = - \sum {I\log (O) + (1 - I)\log (1 - O)} \end{aligned}$$

MS-SSIM loss function can be described as

17 $$\begin{aligned} {L_{MS - SSIM}} = 1 - MS - SSIM(I,O) \end{aligned}$$

TV loss function can be described as

18 $$\begin{aligned} {L_{TV}} = \sum \limits _{i,j} {(({{(F(i,j - 1) - F(i,j))}^2} + {{(F(i + 1,j) - F(i,j))}^2}} ) \end{aligned}$$

where *I* represents the input image and *O* represents the output image. $SSIM( \cdot )$ represents multi scale structural similarity operation. *F*(*i*, *j*) is the pixel value at point (*i*, *j*).

**Fig. 3:**
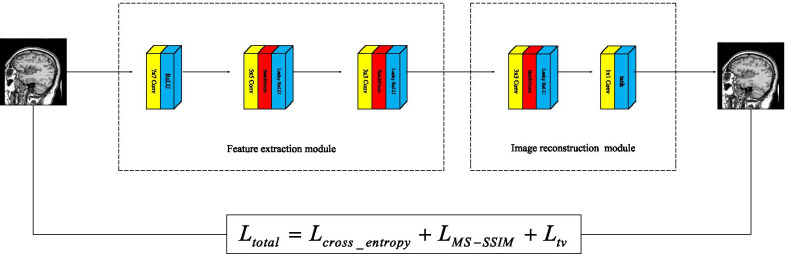
The evolution of images through feature extraction part and image reconstruction part

As shown in Fig. 3, our images reduce the loss of much information through the feature extraction layer and the image reconstruction layer.

### Dataset

We use the brain medical images provided by the Harvard Medical School website as the data set. The data set of this website consists of normal brain images, stroke images, brain tumor images and brain images of other diseases. In order to enhance the robustness of our model, we selected 8000 images from the normal and abnormal images on the website as the training images of our model. All images have a resolution of $256 \times 256$ and are saved in the training set in Portable Network Graphics (PNG) format. The data set contains both CT and MRI, which are good at expressing structure, as well as PET and SPECT, which are professional at expressing metabolic information.

### Qualitative and quantitative analysis methods

Qualitative methods are to combine the knowledge of medical nuclear imaging to qualitatively analyze our fusion images. The quality of brain image fusion is judged by the texture, edge and color of the image; Quantitative methods are to use several representative image fusion evaluation metrics to evaluate the quality of brain image fusion. Evaluation metrics are generally divided into five categories. There are evaluation metrics based on information theory, mainly including information entropy, mutual information, edge mutual information and normalized mutual information; there are evaluation metrics based on structural similarity, mainly including SSIM [26], MS_SSIM, MSE; there are evaluation metrics based on image features, Mainly include Spatial Frequency (SF), Standard Deviation (SD), Average Gradient (AG); there are evaluation metrics based on source image and generated image, mainly including Correlation Coefficient (CC) and $Q_{AB}^F$ [27]; there are evaluation metrics based on human visual perception, mainly including Visual Information Fidelity (VIF) [28]. In this article, we used six metrics of Cross Entropy (CE), Feature Mutual Information (FMI) [29], SSIM, SF, VIF and $Q_{AB}^F$ to evaluate the quality of our model and comparison algorithm.

## Results

### Experiment details and result analysis

We selected multi-modal medical images of four diseases from the Harvard Medical School website to verify the performance of our model. The four diseases are Glioma, Huntington’s disease, Metastatic bronchogenic carcinoma and Alzheimer’s disease. For structural images, we generally choose MR-T2 images that are good at expressing lesion information; For functional images, we choose SPECT-Tl images, SPECT-Tc images and FDG-PET images. Our model is implemented in the PyTorch framework and trained and tested on a platform with Intel Core i7-8700k CPU and NVIDIA RTX 2080 GPU. In this experiment, we plan to adopt eight representative image fusion algorithms that are mentioned in Background.

#### Glioma

**Fig. 4:**
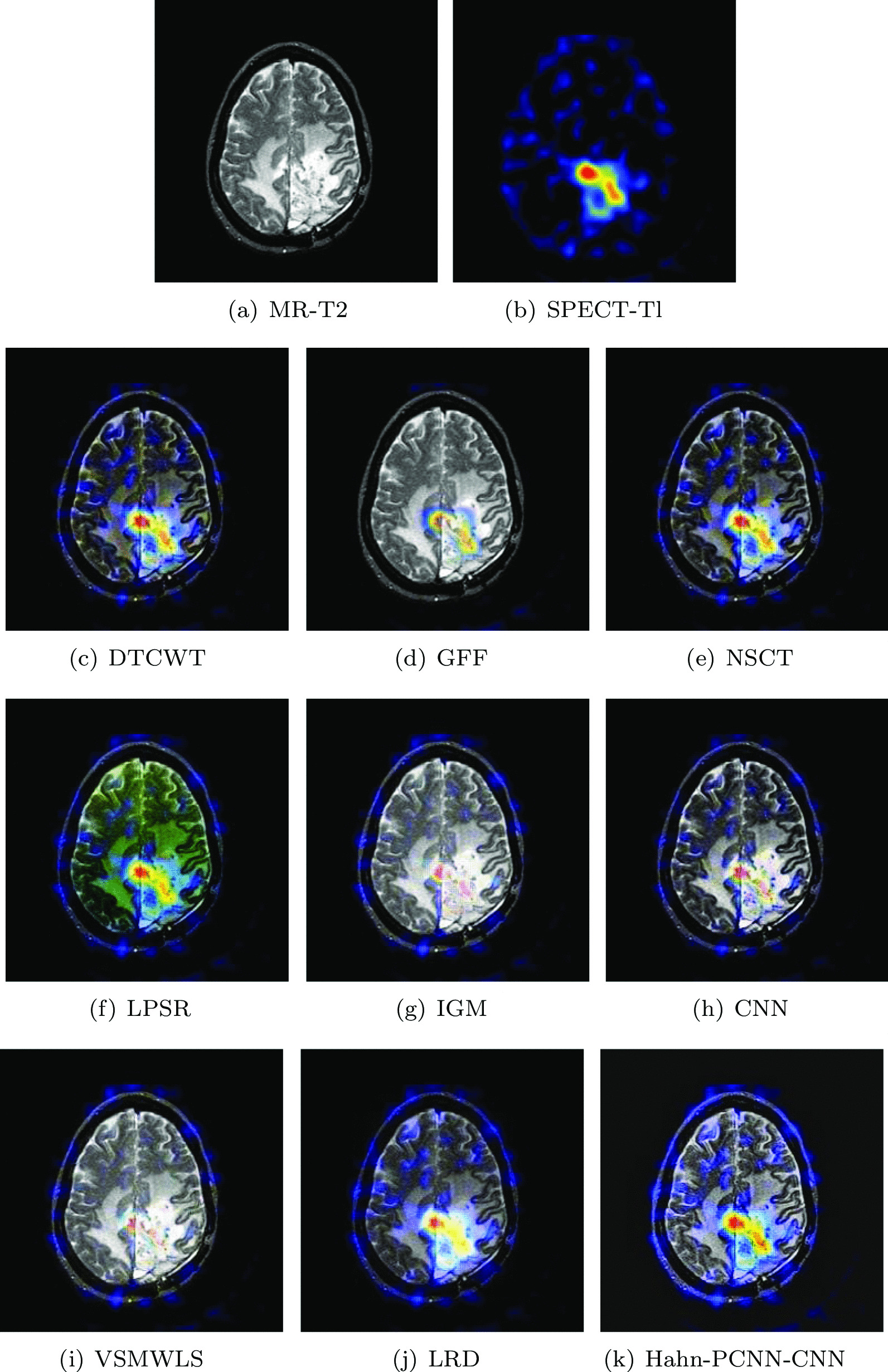
The first set of fused MRI-SPECT images from 9 methods on glioma

**Fig. 5:**
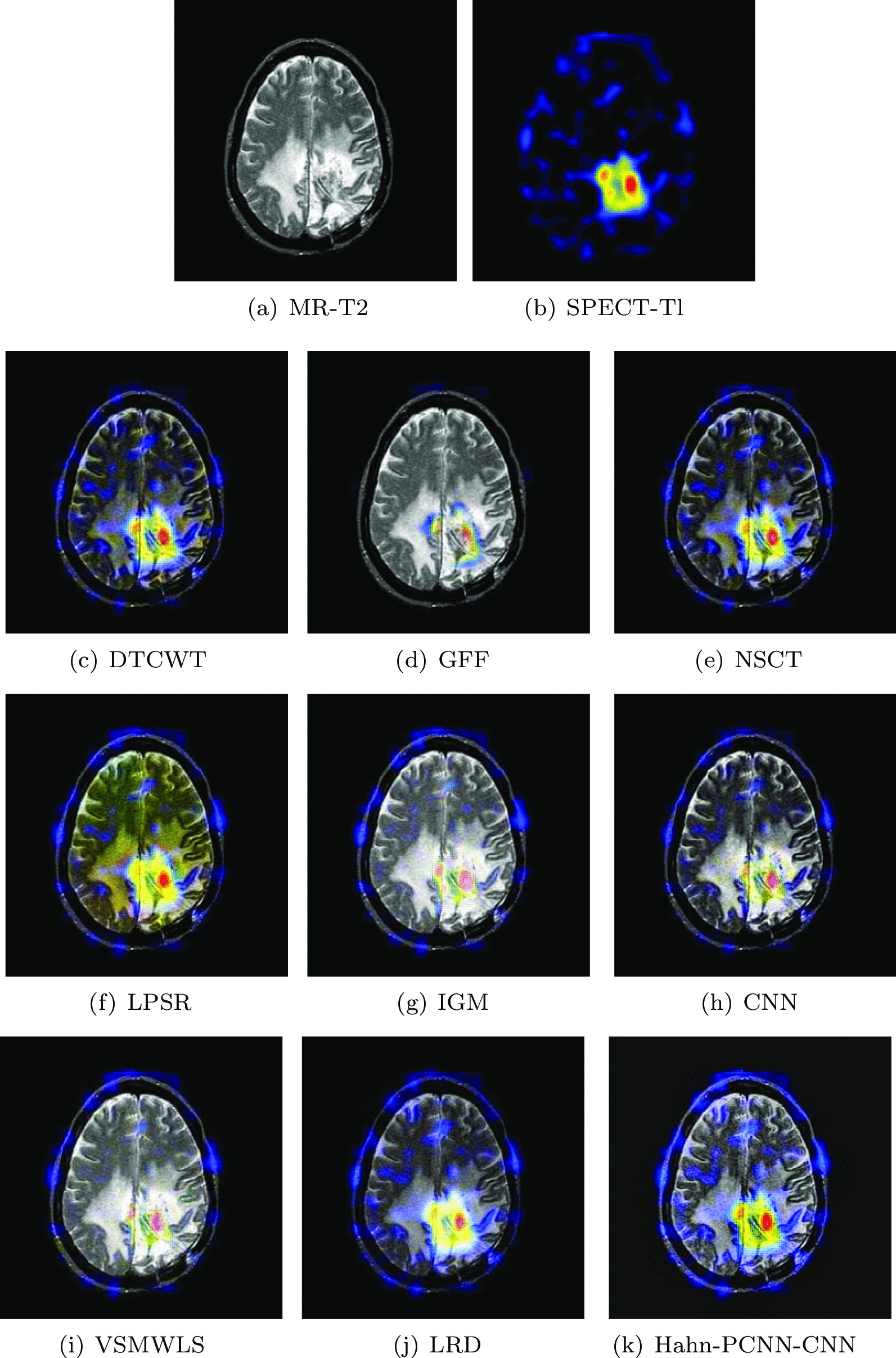
The second set of fused MRI-SPECT images from 9 methods on glioma

The source images in Figs. 4 and 5 are from a 51-year-old female. She has right hemiplegia and hemianopia due to glioma. In the MR-T2 image, the astrocytoma is located in the left parietal lobe; in the SPECT-T1 image, its metabolism is abnormal. Ideally, the fused image should contain both clear astroglioma texture and edges, as well as its metabolism. From Figs. 4 and 5, it can be seen that the fused images obtained based on GFF, IGM and VSMWLS algorithms have better structural information, however, the metabolic state of the tissue can not be obtained. The contrast of the fused image obtained by DTCWT and NSCT algorithms is too low, which causes the image to darken and is not suitable for observation. The fused image obtained based on the CNN algorithm has good structural information and metabolic status, however, the texture and edges of astrogliomas are poor. The fused image obtained by the LPSR algorithm is seriously distorted. Although the fusion image obtained based on the LRD algorithm retains the color information and structural information of the source image well, it is seriously blurred in the diseased tissue, which is not conducive to the diagnosis of the doctor. In general, whether it is normal tissue or glioma, the fused image obtained by our algorithm is the best in preserving structural information and metabolism. Tables 3 and 4 show the quantitative analysis of all fusion algorithms. Obviously, our algorithm performs well in terms of feature mutual information, spatial frequency and cross entropy. Among other metrics, it is also slightly better than other algorithms.

**Table 3:** The objective evaluation scores about group 1 fused images

Methods	Metrics					
	$Q_{AB}^F$	SSIM	VIF	FMI	SF	CE
DTCWT	0.5983	0.5995	0.5663	0.6014	18.3195	0.3081
GFF	0.6807	0.5716	0.6189	0.6606	18.9207	0.2167
NSCT	0.6217	0.6449	0.5828	0.6043	18.3654	0.3141
LPSR	0.5930	0.6291	0.5801	0.6321	17.6417	0.2455
IGM	0.6604	0.5764	0.6663	0.6382	18.9412	0.2191
CNN	0.6480	0.6541	0.5962	0.6455	17.8180	0.2376
VSMWLS	0.5564	0.5222	0.5818	0.6087	14.6674	0.2961
LRD	0.6567	0.5750	0.6498	0.6339	18.9175	0.2257
Hahn-PCNN-CNN	0.6838	0.7792	0.6671	0.6852	22.8255	0.1972

**Table 4:** The objective evaluation scores about group 2 fused images

Methods	Metrics					
	$Q_{AB}^F$	SSIM	VIF	FMI	SF	CE
DTCWT	0.5850	0.5655	0.5233	0.6019	18.4431	0.3297
GFF	0.6766	0.5474	0.5467	0.6716	19.2020	0.2267
NSCT	0.6106	0.6139	0.5479	0.6243	18.5155	0.3095
LPSR	0.6255	0.6220	0.6128	0.6509	18.6395	0.2609
IGM	0.6362	0.5740	0.6145	0.6513	18.9933	0.2218
CNN	0.6334	0.6370	0.5678	0.6536	21.8355	0.2574
VSMWLS	0.5387	0.4918	0.5495	0.6167	14.7282	0.3167
LRD	0.6381	0.5650	0.5957	0.6455	19.0145	0.2178
Hahn-PCNN-CNN	0.6967	0.6438	0.6984	0.6950	23.1363	0.1705

#### Huntington’s disease

**Fig. 6:**
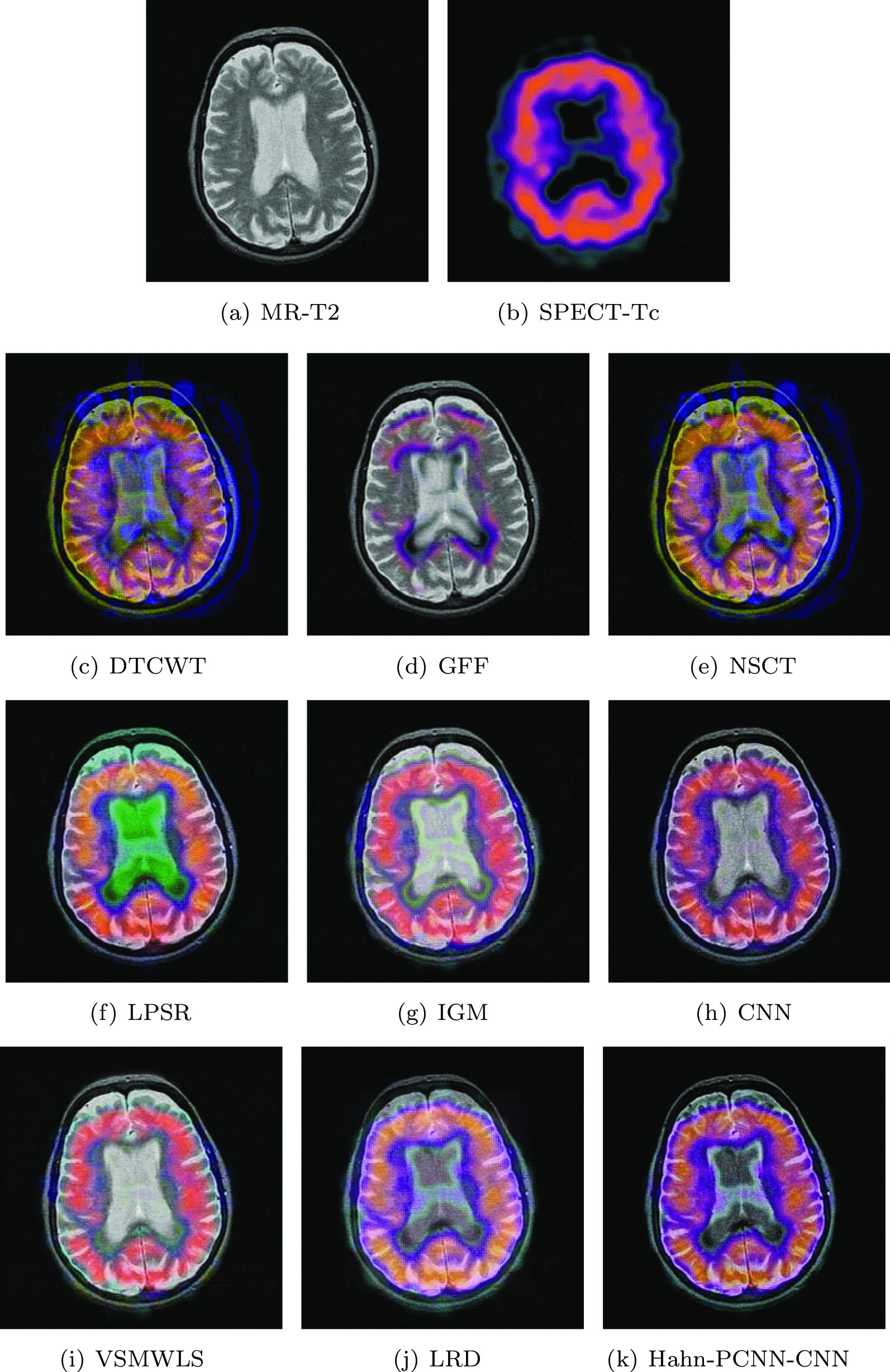
The first set of fused MRI-SPECT images from 9 methods on huntington’s disease

**Fig. 7:**
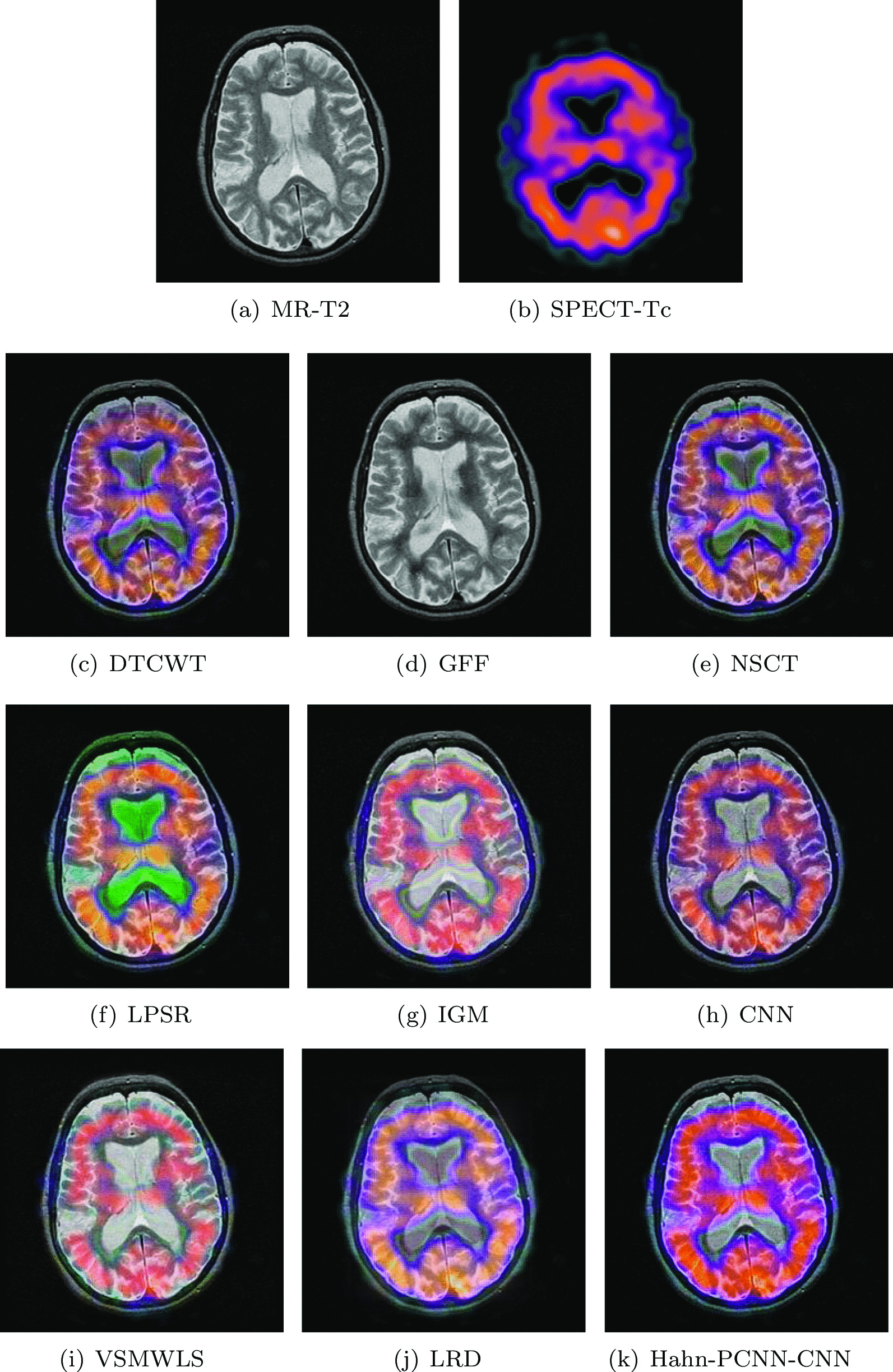
The second set of fused MRI-SPECT images from 9 methods on huntington’s disease

The source images in Figs. 6 and 7 are from a 70-year-old woman who was clinically diagnosed as Huntington’s disease. In the MR-T2 image, the caudate nucleus has a tendency to shrink ; in the SPECT-Tc image, we can see the metabolism of the caudate nucleus in the shrinking process. Therefore, the fusion of the above two images has far-reaching significance. The structure information of the fusion image obtained by GFF, IGM and VSMWLS algorithms is well preserved, but the metabolism can hardly be expressed; the color information of the fusion image obtained based on the LPSR algorithm is seriously distorted; the fusion image obtained based on the LRD algorithm contains the source image. However, the detail information is seriously lost; the fused image obtained by DTCWT and NSCT algorithms can also obtain the source image information, however, the visual fidelity is somewhat poor and the low contrast leads to dark shadows in the caudate body; the fused image obtained by the GFF algorithm has a deviation in the expression of metabolic information at the edge of the caudate nucleus; our model performs well in retaining overall structural information and the expression of metabolic conditions in and around the caudate nucleus. The evaluation results shown in Tables 5 and 6 can prove that our model performs far better than other algorithms on the metric of structural similarity; on other metrics, the model is also quite satisfactory and stable.

**Table 5:** The objective evaluation scores about group 3 fused images

Methods	Metrics					
	*Q*_*AB*_^*F*^	SSIM	VIF	FMI	SF	CE
DTCWT	0.3861	0.4348	0.4464	0.2852	17.4331	0.8959
GFF	0.5720	0.6468	0.4506	0.3532	18.9569	0.8728
NSCT	0.3976	0.4788	0.4817	0.2887	17.4846	0.8479
LPSR	0.5423	0.7266	0.5427	0.3284	18.9312	0.8081
IGM	0.4853	0.5885	0.5013	0.3227	17.8907	0.8875
CNN	0.5141	0.6261	0.4613	0.2933	19.6962	0.8215
VSMWLS	0.4470	0.6027	0.5735	0.3037	15.0761	0.8140
LRD	0.5102	0.6293	0.5016	0.3008	18.0826	0.6713
Hahn-PCNN-CNN	0.5731	0.7636	0.5957	0.3741	19.8026	0.6092

**Table 6:** The objective evaluation scores about group 4 fused images

Methods	Metrics					
	*Q*_*AB*_^*F*^	SSIM	VIF	FMI	SF	CE
DTCWT	0.5203	0.5950	0.4491	0.3454	19.9903	0.8249
GFF	0.3054	0.5114	0.4355	0.2843	13.1245	0.4720
NSCT	0.5372	0.6469	0.4810	0.3594	20.1095	0.7836
LPSR	0.5487	0.6896	0.5029	0.3719	22.3260	0.3817
IGM	0.5076	0.5443	0.4583	0.3873	19.1917	0.5209
CNN	0.5345	0.6054	0.4381	0.3562	21.2160	0.7761
VSMWLS	0.5085	0.5873	0.4587	0.3261	19.2965	0.5004
LRD	0.4187	0.5584	0.5363	0.2913	15.2035	0.5092
Hahn-PCNN-CNN	0.5498	0.7878	0.5387	0.3891	22.3293	0.3802

#### Metastatic bronchogenic carcinoma

**Fig. 8:**
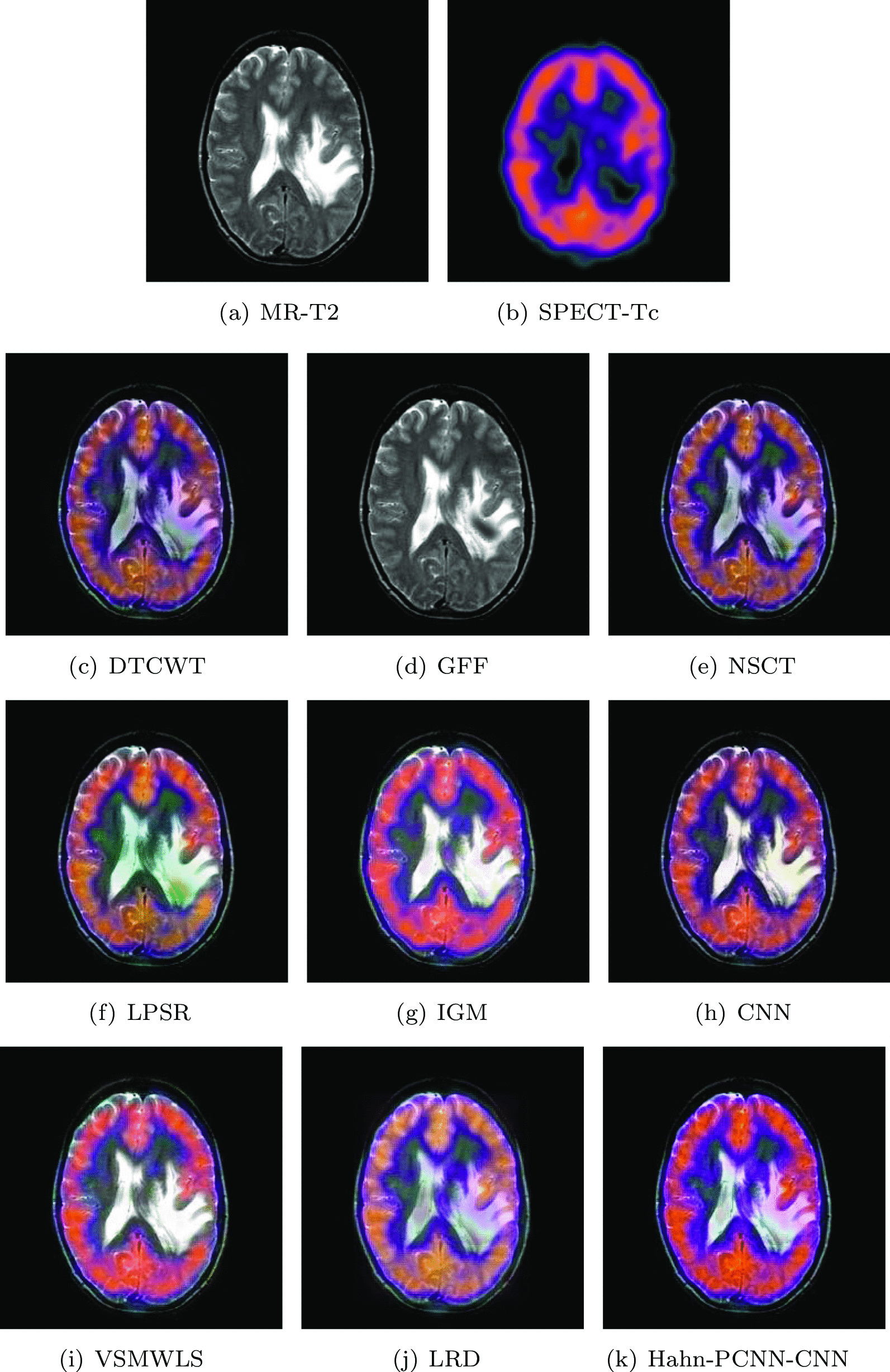
The first set of fused MRI-SPECT images from 9 methods on metastatic bronchogenic carcinoma

**Fig. 9:**
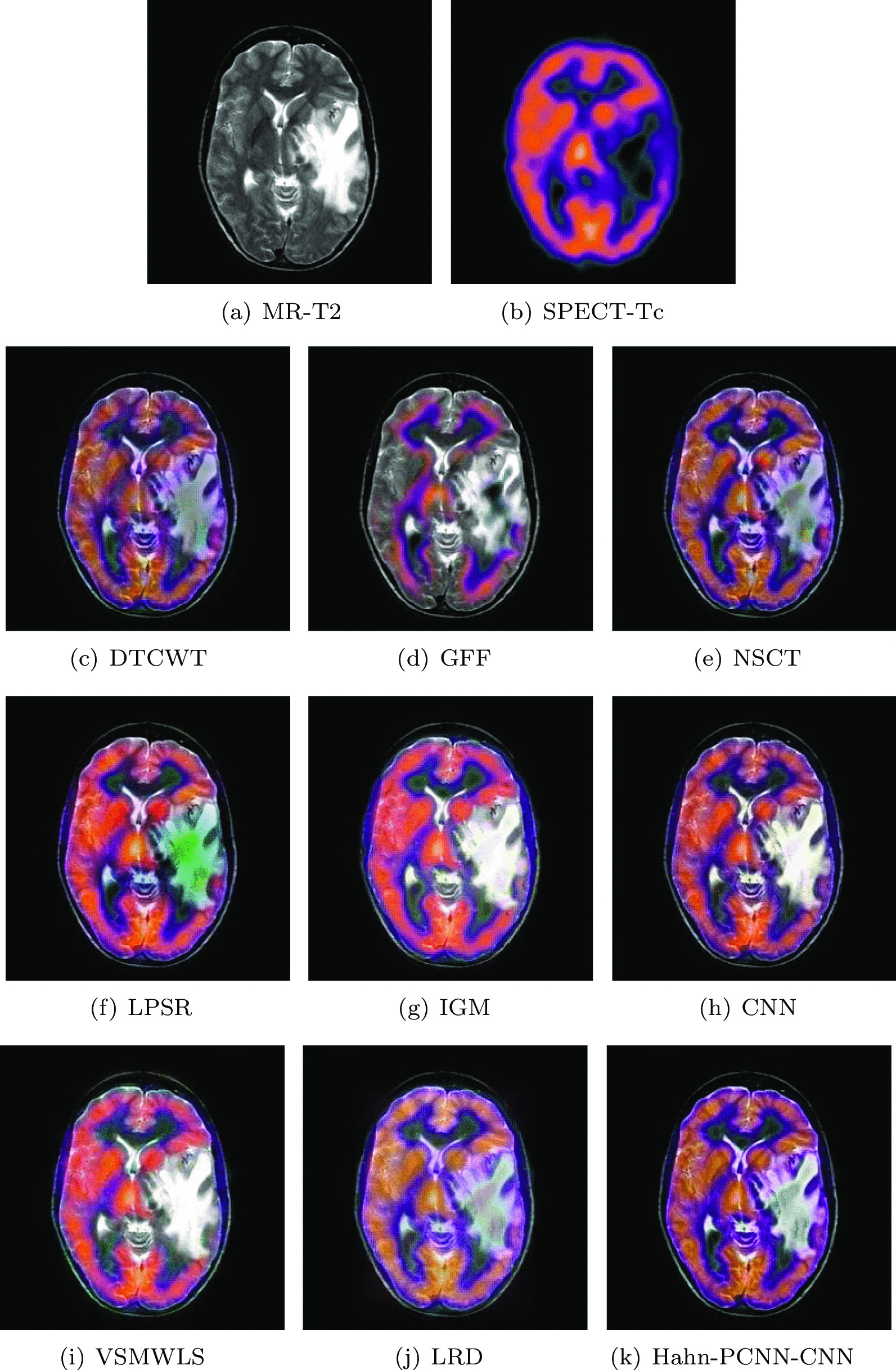
The second set of fused MRI-SPECT images from 9 methods on metastatic bronchogenic carcinoma

The source images in Figs. 8 and 9 are from a 42-year-old patient with brain metastases from bronchial cancer. In the MR-T2 image, most of the left temporal area showed high signal intensity. The narrow sulcus in the left hemisphere is caused by severe swelling of the left hemisphere; in the SPECT-Tc image, the blood flow in the lesion area is very low. Ideally, the fused image can not only maintain high signal strength and the metabolic status of the diseased area, but also eliminate interference information. Algorithms like IGM, CNN and VSMWLS, the overall information of the fused image is well preserved, but the metabolism of the lesion is almost absent. The fused image obtained based on the GFF algorithm shows inexplicable black blocks at the lesion; the fused image obtained by DTCWT and NSCT algorithms retain the metabolism of the tissue, but the edge structure is blurred in details; the color is distorted during the fusion process based on the LPSR algorithm; the fused image obtained based on the LRD algorithm can present a better metabolic status, but the overall texture information is still unclear. The fused image obtained by our model performs well in anatomical information retention, metabolic information acquisition and visual perception. In Tables 7 and 8, we found that our algorithm excels in cross entropy, structural similarity and spatial frequency and is better than other algorithms in other metrics.

**Table 7:** The objective evaluation scores about group 5 fused images

Methods	Metrics					
	$Q_{AB}^F$	SSIM	VIF	FMI	SF	CE
DTCWT	0.5338	0.6173	0.5345	0.6390	17.2110	0.3243
GFF	0.3500	0.5700	0.5854	0.6503	11.4905	0.3959
NSCT	0.5462	0.6766	0.5852	0.6610	17.2561	0.2981
LPSR	0.5702	0.7618	0.6424	0.6979	17.5615	0.3264
IGM	0.4685	0.7007	0.6266	0.6495	15.8747	0.3751
CNN	0.5328	0.7324	0.6194	0.6919	18.0280	0.2577
VSMWLS	0.5346	0.7416	0.6399	0.6773	16.9311	0.4165
LRD	0.4600	0.6851	0.6830	0.6243	13.7973	0.3925
Hahn-PCNN-CNN	0.5732	0.8464	0.6848	0.6994	18.0290	0.2560

**Table 8:** The objective evaluation scores about group 6 fused images

Methods	Metrics					
	$Q_{AB}^F$	SSIM	VIF	FMI	SF	CE
DTCWT	0.5162	0.5854	0.4655	0.6069	18.7235	0.3018
GFF	0.5660	0.6326	0.4165	0.6917	19.0711	0.2555
NSCT	0.5340	0.6635	0.5234	0.6459	18.9222	0.2639
LPSR	0.5376	0.7405	0.5835	0.6782	19.0237	0.2111
IGM	0.4752	0.6564	0.5545	0.6460	17.1588	0.3222
CNN	0.5224	0.7132	0.5494	0.6745	20.2132	0.3133
VSMWLS	0.4662	0.6400	0.6091	0.6133	15.5493	0.3542
LRD	0.5128	0.6845	0.5666	0.6559	18.0514	0.3483
Hahn-PCNN-CNN	0.5671	0.7597	0.6209	0.6926	21.6100	0.1755

#### Mild Alzheimer’s disease

**Fig. 10:**
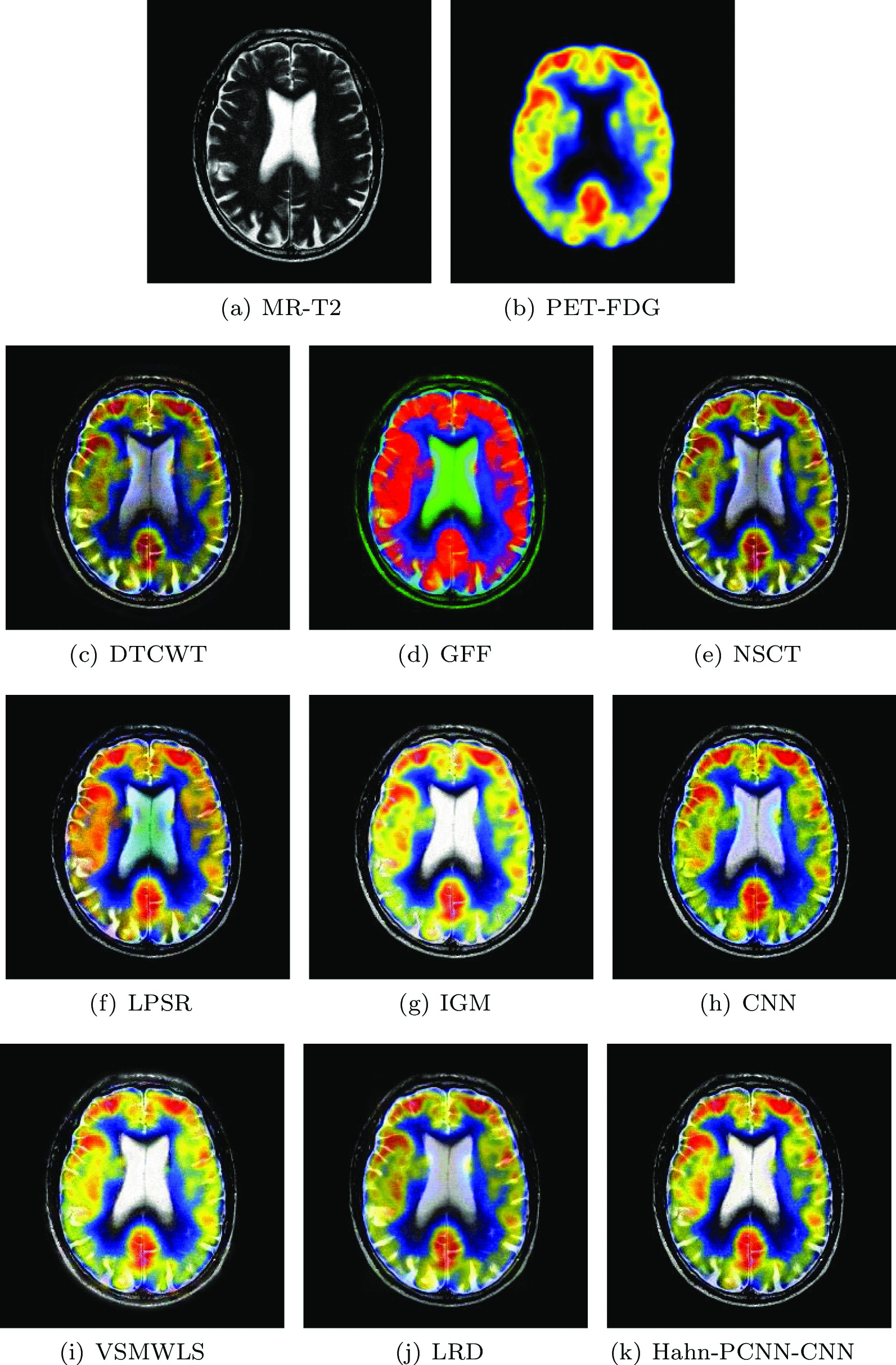
The first set of fused MRI-PET images from 9 methods on mild Alzheimer’s disease

**Fig. 11:**
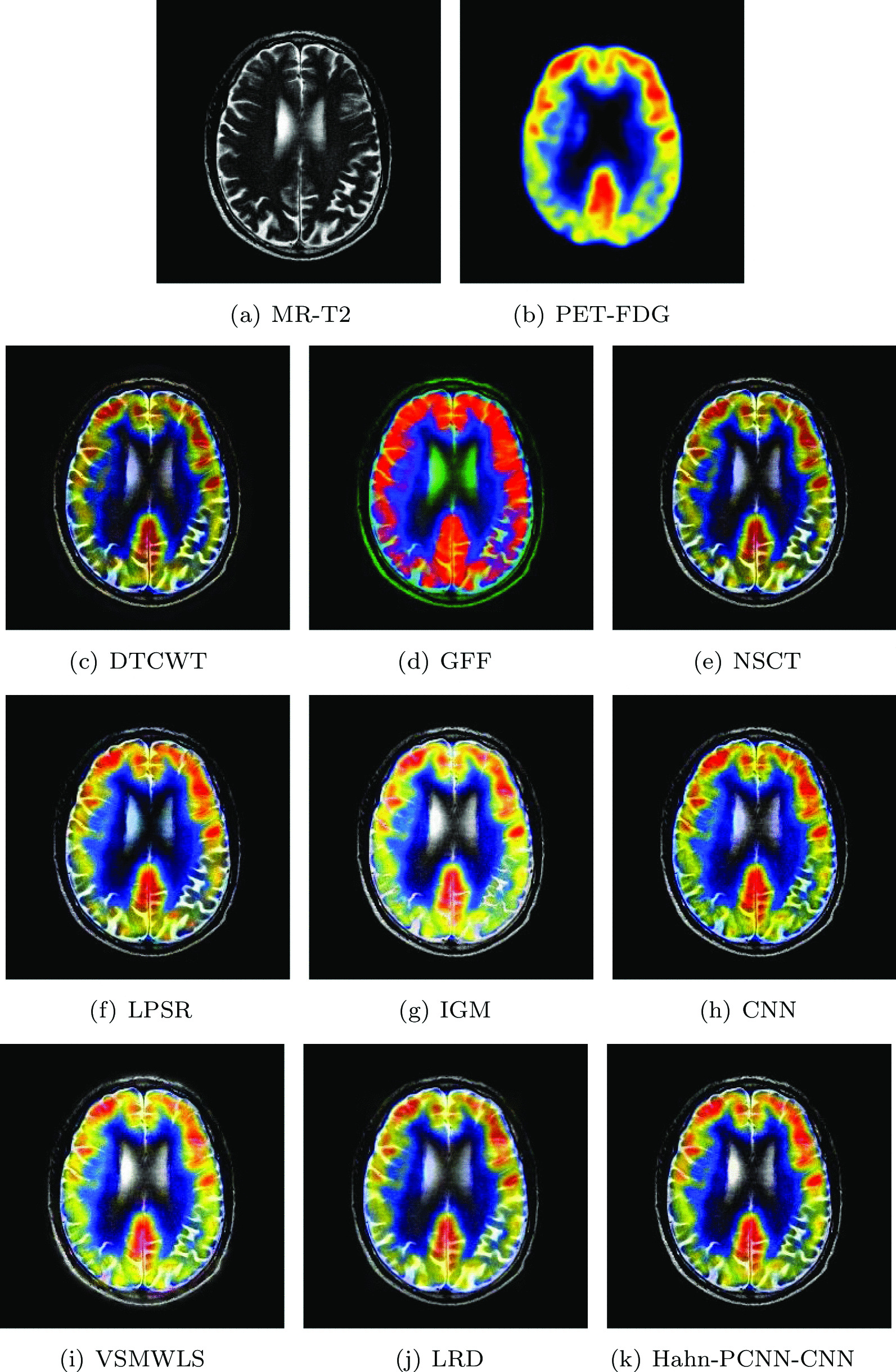
The second set of fused MRI-PET images from 9 methods on mild Alzheimer’s disease

The source images in Figs. 10 and 11 are from a 70-year-old man. He suffers from memory loss and is always depressed, which is a manifestation of mild Alzheimer’s disease. In the MR-T2 image, the global hemispheric sulcus is enlarged and is more prominent in the parietal lobe; in PET-FDG images, local brain metabolism is abnormal, especially in the anterior and posterior regions of the parietal wall of the temporal lobe. Changes have also occurred on both sides of the temporal lobe. Ideally, the above-mentioned information is included in the fused image and at the same time, interference information items are eliminated. The color distortion of the fused image obtained based on GFF and LPSR algorithms will affect the expression of metabolism; the brightness of the fused image obtained by IGM and VSMWLS algorithms is too high, resulting in unclear structural information; the brightness of the fused image obtained based on DTCWT and NSCT algorithms is too dark, resulting in unclear edges of the caudate nucleus; the fused image obtained by CNN and LRD algorithms retain the effective information of the source image, but the color saturation is poor; the fused image obtained based on the Hahn-PCNN-CNN algorithm realizes the perfect fusion of the source image, both in terms of structural information and metabolism. At the same time, it also performs well in terms of chroma and saturation and the image is easy to observe. Tables 9 and 10 show the objective performance of all algorithms. It can be seen that the Hahn-PCNN-CNN algorithm is superior to other algorithms in all metrics.

**Table 9:** The objective evaluation scores about group 7 fused images

Methods	Metrics					
	$Q_{AB}^F$	SSIM	VIF	FMI	SF	CE
DTCWT	0.4743	0.4454	0.3624	0.5655	26.9326	0.3822
GFF	0.3679	0.4612	0.3108	0.6074	18.9252	0.3541
NSCT	0.5178	0.5147	0.3878	0.6229	27.4106	0.3529
LPSR	0.4999	0.5338	0.3964	0.6612	27.0963	0.2105
IGM	0.5383	0.6012	0.4251	0.6701	27.2623	0.2107
CNN	0.5220	0.5769	0.4085	0.6741	20.5773	0.1924
VSMWLS	0.4653	0.5358	0.4087	0.6226	25.9969	0.1941
LRD	0.4760	0.4782	0.4495	0.6126	22.1965	0.2633
Hahn-PCNN-CNN	0.5393	0.6065	0.4502	0.6762	28.7803	0.1904

**Table 10:** The objective evaluation scores about group 8 fused images

Methods	Metrics					
	$Q_{AB}^F$	SSIM	VIF	FMI	SF	CE
DTCWT	0.4956	0.5142	0.4285	0.5815	27.7145	0.2668
GFF	0.3633	0.4777	0.3782	0.6199	18.9824	0.2818
NSCT	0.5272	0.5711	0.4690	0.6300	27.9576	0.2664
LPSR	0.5106	0.5851	0.4831	0.6650	27.5567	0.1360
IGM	0.5035	0.6061	0.5048	0.6880	26.8727	0.1503
CNN	0.5217	0.5854	0.4925	0.6882	28.3076	0.1276
VSMWLS	0.4620	0.5632	0.4911	0.6429	26.3168	0.1404
LRD	0.4724	0.5528	0.5356	0.6444	22.4599	0.1699
Hahn-PCNN-CNN	0.5292	0.6297	0.5370	0.6888	28.6091	0.1251

### Comparison and analysis with IFCNN

Inspired by Image Fusion Framework based on Convolutional Neural Network (IFCNN), we proposed our model. In multi-modal brain medical image fusion, IFCNN can not fuse images containing metabolic information of organs and tissues and only performs image fusion on CT and MRI. Therefore, the functional image with color information can not be directly fused in IFCNN, however, our model can do it. In the process of model training, we added color images to the training set so that our model can fully process this type of image. In order to prove that our model is more suitable for multi-modal brain medical image fusion than IFCNN, we designed a set of experiments to convert functional images with colors to three representative color spaces that are RGB, HSV and YUV. Then we compare their fusion results with ours.

**Fig. 12:**
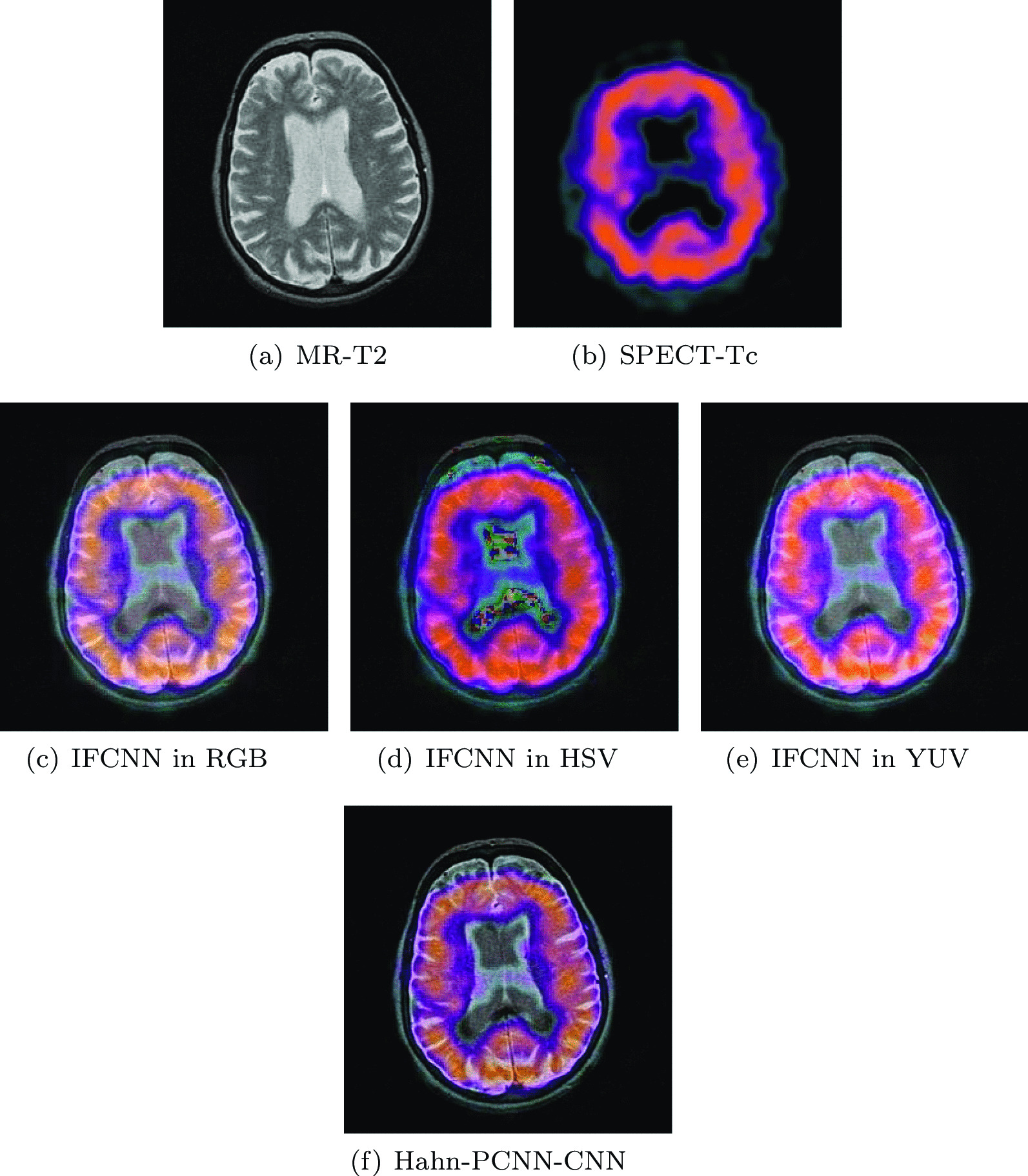
The second group of source images and fused images by IFCNN in different color space and Hahn-PCNN-CNN

In Fig. 12, we can clearly find that the final images of the three color spaces obtained by IFCNN are not as good as our model. In HSV color space and YUV color space, the fused images have poor texture and blur and it is difficult to provide valuable information to doctors. In RGB color space, the fused image has better texture information preservation, but it lags far behind the fused image generated by our model in terms of visual fidelity. Therefore, in the multi-modal brain medical image fusion, our model is far superior to IFCNN.

### Time complexity analysis

**Fig. 13:**
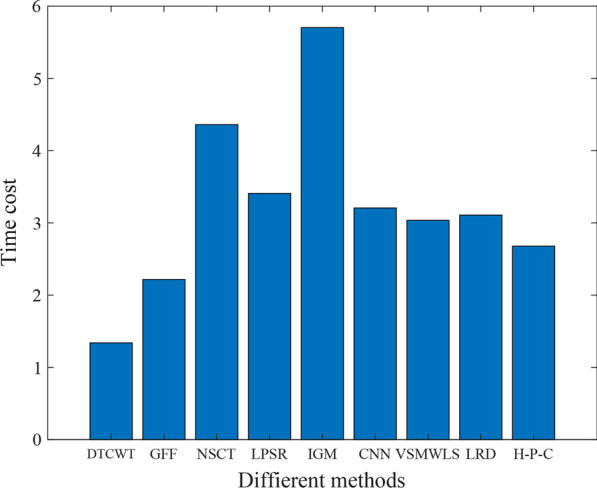
The time complexity of different types of multi-modal medical images

In this part, we focus on the time complexity of our model. Time complexity is an important metric that measures the efficiency of a model in this field. Generally, time complexity is directly proportional to the fusion effect. That is, the more time it takes, the better the result of the fusion. However, this is not the result we want. Our goal is to obtain a better fusion result in a short time through our fusion algorithm. In the article, we conducted a total of eight sets of multi-modal brain medical image fusion experiments.We calculate the average time loss of eight sets of images under different algorithms and put it as the average time loss of the corresponding algorithm. Figure 13 shows the average time loss of all algorithms.

Figure 13 shows the average time loss (in seconds) of all algorithms. Among the above methods, DTCWT showed the smallest average execution time, while IGM showed the highest execution time. Considering our proposed Hahn-PCNN-CNN algorithm, compared with NSCT, LPSR, CNN, VSMWLS, LRD, its average execution time is shorter; compared with GFF, its average execution time is longer. This is because our model uses the block potential of Hahn moment blocks, thus avoiding the possibility of image artifacts and enhancing visualization. Therefore, the algorithm execution time will increase, resulting in an average time consumption higher than the average time consumption of DTCWT and GFF. It can be seen that the focus of future work is how to minimize the average time loss of the model.

### Statistical analysis of the results

**Fig. 14:**
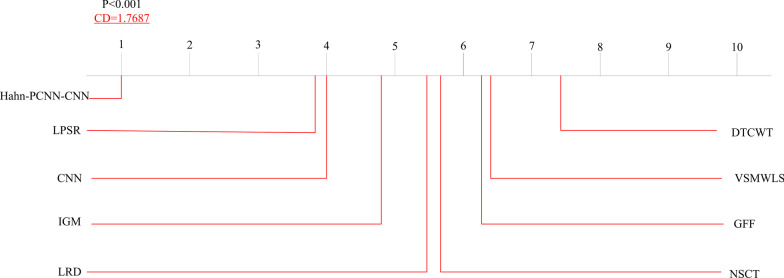
The time complexity of different types of multi-modal medical images

We used a non-parametric test method to verify the performance of our model. Among the many non-parametric testing methods, Friedman’s test can make full use of all the information in the relevant samples. Others such as Kruskal-Wallis test and the popular median method are also correct to judge whether multiple related samples come from a population with significant differences in size, but this approach is like using a non-parametric method to test two independent samples. The information about the differences between different individuals in the sample is ignored, thereby reducing the power of the test. Therefore, We used the non-parametric Friedman’s test and the post-hoc Nemenyi test to analyze how the analyzed methods differ from each other. Tukey’s distribution is introduced to calculate the Critical Difference (CD). If the difference between the algorithm levels is greater than the CD, the difference is considered significant [30]. We use the values to calculate the level of the average data fusion method from Tables 3, 4, 5, 6, 7, 8, 9 and 10. The resulting grade is shown in Fig. 14.

In Fig. 14, we find that the level difference between all comparison algorithms and our algorithm is greater than the value of CD. Therefore, our algorithm has obvious statistical advantages compared with other comparison algorithms.

### Tri-modal fusion

Brain medical images are often different from other types of images. Since the imaging comes from multiple sensors, each sensor has a different focus. CT images focus on skill tissues; MRI images have better imaging effect on brain tissues; PET images help doctors observe the metabolism state of tissues. Therefore, it is very challenging to extend the traditional fusion method of two-mode medical images to three or more images while avoiding the overlap of information and the blurring of key textures. Such research is of great value. Most fusion methods are designed for image fusion of two modes. If you want to achieve tri-modal image fusion, you can only manually select two images for fusion and then merge the fusion result with the third image to get the final result. This is not the best choice because the fusion algorithm assumes that the image prior may not appear in the intermediate fusion results. The previous neural network methods all fuse two images from the training set and continuous fusion will result in multiple false images.

**Fig. 15:**
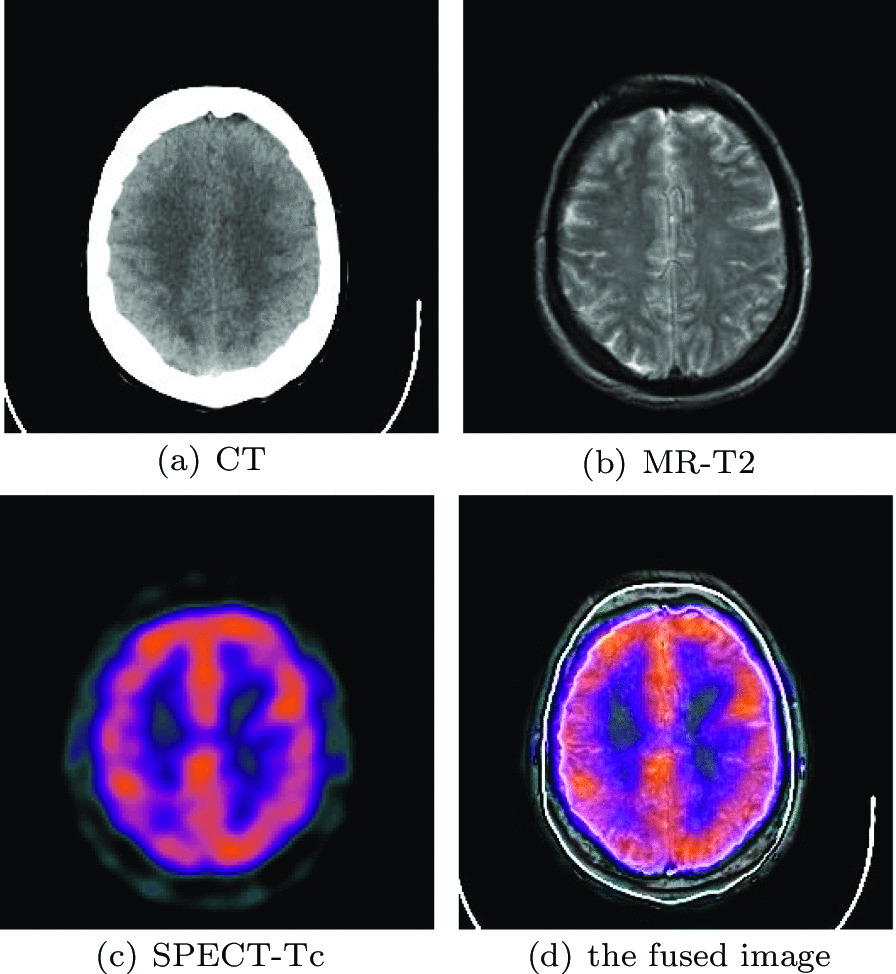
Tri-modal fusion of magnetic resonance imaging, computed tomography, and single-photon emission computed tomography slices. Best viewed on screen

Our fusion model just overcomes shortcomings of the above fusion method. There will be no continuous fusion of multiple false images, nor will it affect the effect of later fusion image because of different selection order in the early stage. Please note that the addition of modalities will not affect the processing time of our neural network because feature maps are extracted in parallel. Figure 15 shows us such an example. In the final fusion image, there are not only the eye-catching skull from CT, but also the clear brain tissue from MRI and metabolic information from PET around it.

## Conclusions

Current research shows that the image obtained by our model retains the texture information of the anatomical image and the color information of the functional image to the greatest extent, while maintaining the contrast of the image during fusion. On the six representative evaluation metrics, the performance of our model is significantly better than the other eight algorithms. In addition, the diversity of images enhances the robustness of our algorithm. Since our algorithm is a lightweight and high-quality algorithm, it has broad application prospects in intelligent medicine. If it is extended to other image data sets, the performance of the model can be improved.

## Data Availability

The datasets used and/or analyzed during the current study are available from http://www.med.harvard.edu/AANLIB/home.html. Experimental images in Fig. 1 are downloaded from http://www.med.harvard.edu/AANLIB/cases/case28/mr1-tc1/010.html; Experimental images in Fig. 2 are downloaded from the 85th coronal slice from http://www.med.harvard.edu/AANLIB/cases/caseNA/pb9.htm; Experimental images in Fig. 3 are downloaded from the 42th sagittal slice from http://www.med.harvard.edu/AANLIB/cases/caseNA/pb9.htm; Experimental images in Fig. 4 are downloaded from http://www.med.harvard.edu/AANLIB/cases/case1/mr1-tl4/038.html; Experimental images in Fig. 5 are downloaded from http://www.med.harvard.edu/AANLIB/cases/case1/mr1-tl4/037.html; Experimental images in Fig. 6 are downloaded from http://www.med.harvard.edu/AANLIB/cases/case11/mr1-tc1/012.html; Experimental images in Fig. 7 are downloaded from http://www.med.harvard.edu/AANLIB/cases/case11/mr1-tc1/011.html; Experimental images in Fig. 8 are downloaded from http://www.med.harvard.edu/AANLIB/cases/case28/mr1-tc1/013.html; Experimental images in Fig. 9 are downloaded from http://www.med.harvard.edu/AANLIB/cases/case28/mr1-tc1/010.html; Experimental images in Fig. 10 are downloaded from http://www.med.harvard.edu/AANLIB/cases/caseNN1/mr1-dg1/015.htm; Experimental images in Fig. 11 are downloaded from http://www.med.harvard.edu/AANLIB/cases/caseNN1/mr1-dg1/016.htm; Experimental images in Fig. 12 are downloaded from http://www.med.harvard.edu/AANLIB/cases/case11/mr1-tc1/012.html; Experimental images in Fig. 15 are downloaded from http://www.med.harvard.edu/AANLIB/cases/case21/mr1-tc1/018.html.

## Abbreviations

CT: Computed tomography
MRI: Magnetic resonance imaging
PET: Positron emission tomography
fMRI: Functional magnetic resonance imaging
PCNN: Pulse-coupled neural network
CBF: Cerebral blood flow
18F-FDG: Positron emission tomography labeling glucose with positron nuclide 18F
CNN: Coupled neural network
PD: Proton density
T2: T2-weighted
PNG: Portable network graphics
SSIM: Structural similarity
SF: Spatial frequency
SD: Standard deviation
AG: Average gradient
CC: Correlation coefficient
VIF: Visual information fidelity
CE: Cross entropy
FMI: Future mutual information
MSE: Mean square error
MS_SSIM: Multi-scale structural similarity
DTCWT: Dual-tree complex wavelet transform
NSCT: Nonsubsampled Contourlet
GFF: Guided filtering fusion
LPSR: Laplacian pyramid sparse representation
IGM: Internal generative mechanism
VSMWLS: Visual saliency map and weighted least square
LRD: Laplacian re-decomposition
SPECT: Single-photon emission computed tomography
